# Efficacy of Nd:YAG Laser and Intralesional Triamcinolone Injection Combination Therapy in the Postoperative Management of Keloids

**DOI:** 10.1007/s00266-024-04433-z

**Published:** 2024-10-07

**Authors:** Jun Ho Park, Ji Won Jeong, Ji-Ung Park

**Affiliations:** Department of Plastic and Reconstructive Surgery, SMG-SNU Boramae Medical Center, Seoul National University College of Medicine, 20 Boramae-ro 5-gil, Dongjak-gu, 07061 Republic of Korea

**Keywords:** Keloid, Laser, Triamcinolone injection

## Abstract

**Background:**

Keloids, characterized by protruding scars that extend beyond the original skin damage site, cause significant emotional stress and reduced quality of life. Their exact pathogenesis remains unclear, with various hypotheses including growth factor imbalances and extracellular matrix changes. No single treatment is universally accepted, but multiple modalities like triamcinolone acetonide injection (TAC), laser therapies, and surgery are commonly used.

**Methods:**

This retrospective study involved East Asian patients who underwent keloid scar excision between March 2019 and June 2022. Patients were divided into two groups: one receiving only TAC injections and the other a combination of TAC and Nd:YAG laser therapy. The efficacy of treatments was evaluated using the modified Vancouver Scar Scale (mVSS) and the Patient and Observer Scar Assessment Scale (POSAS), with follow-ups at six and twelve months after operation.

**Results:**

The study involved 111 patients. Both treatment groups showed significant improvements in mVSS and POSAS scores, but the combination therapy group demonstrated a statistically significant improvement in POSAS scores and lower recurrence rates at 12 months compared to the TAC-only group. However, there was no significant difference in patient satisfaction between the groups.

**Conclusion:**

Dual therapy involving TAC injection and Nd:YAG laser treatment was more effective than TAC injection alone for managing keloid scars after surgery. This combination therapy showed better outcomes in preventing keloid recurrence and improving scar status at 12 months after operation, along with significant improvements in patient-reported outcomes.

**Level of Evidence II:**

This journal requires that authors assign a level of evidence to each article. For a full description of these Evidence-Based Medicine ratings, please refer to the Table of Contents or the online Instructions to Authors www.springer.com/00266.

## Introduction

Keloids are characterized by scar protrusion, itching sensation, redness, and extension of lesion beyond the original site of skin damage. These lesions cause undue emotional stress and lower the quality of life of many patients [1, 2]. Unfortunately, the exact pathogenesis of keloids has not yet been clearly elucidated. Many hypotheses have been proposed including increased levels of transforming growth factor (TGF)-*β* and platelet-derived growth factor (PDGF) receptors, changes in the extracellular matrix (ECM) components, dysregulation of collagen turnover, fibroblast proliferation due to mechanical stretch, genetic immune dysfunction, and immune reaction to sebum [3]. However, these hypotheses do not fully explain the development of keloids in every case.

Due to the unclear pathogenesis of keloids, no single, universally accepted treatment exists. Various treatment modalities have been proposed, including intralesional triamcinolone acetonide injection (TAC), ablative and non-ablative laser therapies, compression garments, silicone gel and sheets, radiotherapy, cryotherapy, and surgical excision. Other intralesional therapies like 5-fluorouracil (5-FU) and bleomycin have also recently been introduced. Each of these treatments has its own strengths and limitations, and growing evidence supports a multimodal treatment over a single mode of treatment in the management of keloids [4, 5].

Of these various treatment modalities, intralesional TAC remains the first-line therapy of keloids in many cases [6]. Triamcinolone is known to inhibit fibroblast proliferation and collagen synthesis via many different mechanisms [3]. Side effects apart from injection-related pain include hypopigmentation, skin atrophy, and telangiectasis, but these often resolve without additional treatment [6, 7]. Systemic side effects like Cushing’s syndrome are rare but still warrant attention.

Various laser therapies for keloids have been developed, albeit with mixed evidence. Laser treatment, typically less painful than intralesional injection, is recognized for both its therapeutic and preventive effects in keloid management. Nd:YAG 1064nm non-ablative laser is known to inhibit collagen synthesis in fibroblast cultures, and evidence supports its use in the clinical setting as well [8–10].

In this study, we aimed to assess the clinical safety and efficacy of combination therapy with intralesional TAC and Nd:YAG laser therapy compared with intralesional TAC monotherapy following surgical excision of keloids.

## Materials and Methods

This retrospective review of postoperative keloid management was approved by our institutional review board (IRB No. 20-2023-46). All patients included in this study provided written informed consent one month postoperatively at the outpatient clinic. The study adhered to the principles of the Declaration of Helsinki and its subsequent amendments. Patients gave informed consent for all surgical procedures, wound management, and the potential use of anonymized photographs.

Patients eligible for this study were those who underwent keloid scar excision between March 2019 and June 2022. All participants were East Asians. A clinical assessment by a plastic surgeon confirmed the diagnosis, and patients underwent thorough preoperative examinations. Medical history, current medication, scar history, and previous treatments were recorded for all patients. Exclusion criteria included poor general condition, adjacent skin diseases, and unrealistic expectations. This single-center study involved three experienced plastic surgeons, each with an average of 18 years of experience, using standardized methods for all procedures.

Following keloid scar excision, all patients received an immediate TAC injection (40 mg/mL) at a dose of 0.1 ml per cm^2^ in the operation room. Treatments began three weeks postoperatively, with five sessions scheduled four weeks apart. Between sessions, the use of a silicone gel sheet (Mepiform, Molnlycke Health Care AB, Sweden) was recommended for both groups. In the single therapy group, only TAC injections were administered at a dose of 0.1 ml per cm^2^ at the scar site. In the combination therapy group, the same dosage of TAC was used for injections. After TAC injection, *Q*-switched Nd:YAG laser treatment was administered, involving six passes at a fluence of 11 J/cm2, spot size 3 mm, pulse duration of 10ns, and frequency of 3 Hz.

All evaluation parameters were examined preoperatively and at 6 and 12 months postoperatively, with the 6 month visit occurring one month after the last treatment session. Three blinded third-party physicians graded scars with the modified Vancouver Scar Scale (mVSS), evaluating vascularity, pigmentation, pliability, height, pain, and pruritus, with scores ranging from 0 (best) to 16 (worst imaginable). The same physicians also graded scars with the Patient and Observer Scar Assessment Scale (POSAS), evaluating vascularity, pigmentation, thickness, relief, pliability, and surface area. Patients also graded their own scars with the POSAS, assessing pain, itching, color, stiffness, thickness, and irregularity. Each POSAS category was scored 1 to 10, with the total score ranging from 6 (best) to 60 (worst). For every evaluation measure, the mean for each group was calculated and compared.

Patients also evaluated their satisfaction level on a 5-point scale from 1 (very poor) to 5 (very satisfied) at each timepoint. Patients who experienced pruritus, pain, and significant scar enlargement (more than double the immediate postoperative scar size) were deemed to have recurrent lesions.

Statistical analyses compared assessment scores at each visit using paired *t *test methods, and descriptive statistics were reported as mean values ± standard deviations (range). The normality of data was tested using the Kolmogorov–Smirnov test. Analyses were performed using SPSS Statistics for Windows, version 26.0 (IBM Corp., Armonk, NY, USA). A *p* value of less than 0.05 was considered statistically significant.

## Results

The study included 111 patients, with a mean age of 35.2 years (ranging from 16 to 69 years), and a mean follow-up duration of 13 months. The single therapy group comprised 58 patients, while the combination therapy group included 53 patients. In terms of keloid site, the ear was the most common (*n*=55). Other characteristics are summarized in Table 1.

**Table 1:** Demographic findings of the patients

Criteria	Intralesional Triamcinolone only	Intralesional triamcinolone + ND:YAG laser
Total number of patients	58	53
Mean age (years)	34.8 ± 25.4	35.6 ± 24.7
Mean follow-up period (months)	13.1 ± 0.2	12.9 ± 0.4
Gender		
Male	17	14
Female	41	39
Location		
Ear	28	27
Face	3	3
Chest	9	8
Abdomen	6	5
Back	3	3
Extremity	9	7
Previous treatments		
Triamcinolone injection	44	45
Surgery	11	7
Laser	2	1
Radiotherapy	1	0
Chemotherapy agent	0	0

The average time elapsed between keloid development and surgery was 6.2±11.9 months for the combination therapy group and 6.6±13.7 months for the single therapy group, a difference that was not significant (*p*=0.87, as shown in Table 2). Both groups demonstrated significant improvements in scar scale changes as measured by the mVSS at 6 months (3.6 ± 0.4) and one year (3.5 ± 0.3) after operation. However, the intergroup differences were not statistically significant at any follow-up visit.

**Table 2:** Comparison of outcomes for TAC single therapy and TAC-Nd:YAG laser combination therapy

	Intralesional Triamcinolone only	Intralesional triamcinolone with ND:YAG laser	*P* value
Mean mVSS			
Initial	9.3 ± 0.6	9.1 ± 0.5	0.71
6 months	3.4 ± 0.3	3.6 ± 0.4	0.84
12 months	3.7 ± 0.5	3.5 ± 0.3	0.92
Mean POSAS			
Initial	7.9 ± 1.4	8.2 ± 1.7	0.54
6 months	2.8 ± 0.3	2.6 ± 0.4	0.66
12 months	2.7 ± 0.4	1.9 ± 0.3	< 0.01
Recurrence rate			
6 months	6.25% (3/48)	0% (0/46)	< 0.01
12 months	12.5% (6/48)	4.35% (2/46)	< 0.01
Overall patient satisfaction			
6 months	4.1 ± 0.9	4.3 ± 0.7	0.74
12 months	4.4 ± 0.6	4.5 ± 0.5	0.85

*ND*:*YAG* Neodymium-doped yttrium aluminum garnet, *mVSS* modified Vancouver Scar Scale, *POSAS* Patient and Observer Scar Assessment Scale

Similar trends were observed in the POSAS scores, with both groups showing significant improvements at 6 months and 1 year after operation. Notably, the mean POSAS score was significantly lower in the combination group than in the monotherapy group at 12-month follow-up (1.9 ± 0.3 vs. 2.7 ± 0.4, *p *< 0.01). The recurrence rate was significantly lower with the combination therapy compared to single therapy at each follow-up timepoint. Patient satisfaction was high in both groups, though the difference was not statistically significant. There were no reported complications, such as infection, hematoma, or wound dehiscence, in either group.

**Fig. 1:**
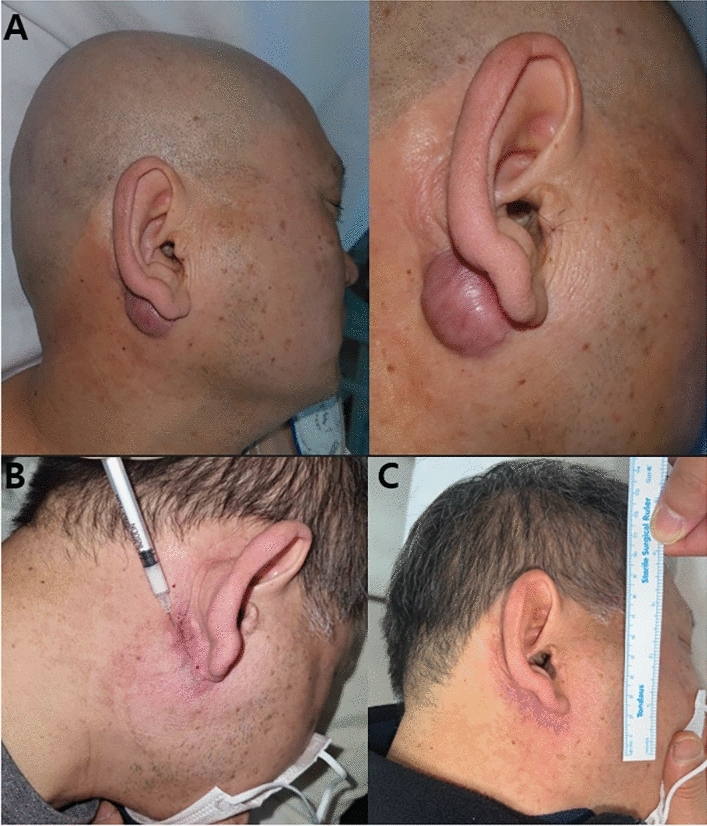
A case of a 58 year-old male patient treated with TAC injection and Nd:YAG laser therapy after surgical excision of keloid. **A** Initial photographic finding, **B** 3 months after surgery, **C** 1 year after surgery

**Fig. 2:**
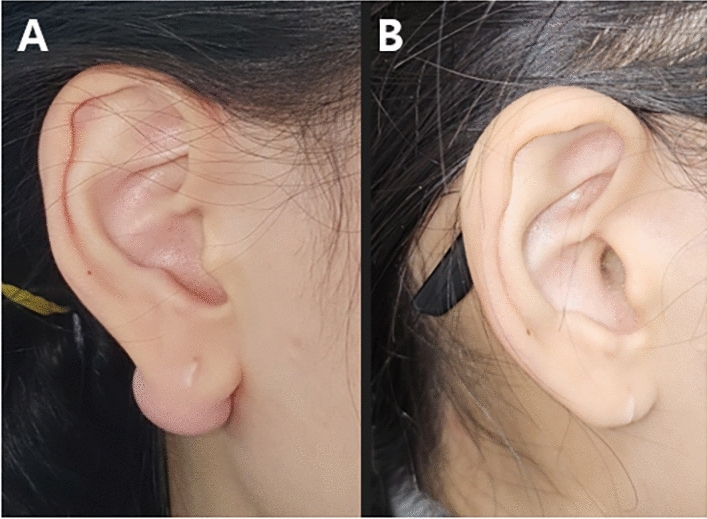
A case of a 34-year-old female patient treated with TAC injection and Nd:YAG laser therapy after surgical excision of keloid. **A** Initial photographic finding, **B** 1 year after surgery

**Fig. 3:**
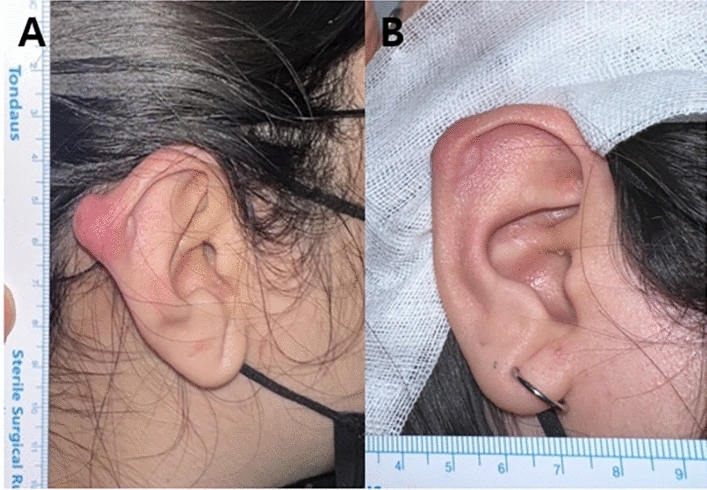
A case of a 18-year-old female patient treated with TAC injection and Nd:YAG laser therapy after surgical excision of keloid. **A** Initial photographic finding, **B** 1 year after surgery

These results suggest that while both single and combination therapies were effective in improving aesthetic outcomes and preventing keloid recurrence, the combination therapy showed superior results in preventing the recurrence of keloid scars and in improving scar status at 12 months after operation. Sample cases of patients treated with combination therapy are highlighted in Figs 1, 2, and 3.

## Discussion

The exact mechanism of keloid development is still largely unknown. Researchers have proposed intriguing hypotheses regarding the pathogenesis of this elusive disorder. Al-Attar et al, in their review of keloid pathogenesis and treatment, summarized some hypotheses of keloid formation [3]. One of them is the altered growth factor levels and ECM composition of keloids in comparison with ordinary scar tissue. Both TGF-beta and PDGF are growth factors that are normally found in wound healing. In keloids, however, the levels of TGF-beta and the receptors of PDGF tend to be elevated and the proliferative effect of these factors are synergistic [11]. The disorganized ECM in keloid tissues is also remarkable. Fibronectin levels are high, whereas hyaluronic acid levels are low [12]. Some proteoglycans such as biglycan and decorin are aberrantly produced in keloids, leading to an abnormal collagen architecture [13]. This disruption in the equilibrium of ECM in turn causes dysregulation of growth factor activity.

The collagen turnover hypothesis focuses on the accumulation of collagen in keloids. The collagen nodule is a representative feature of keloids. Interestingly, not only does excess collagen accumulate in keloid tissue, but the ratio of type I to III collagen is also significantly higher than that of normal tissue. The turnover of collagen is largely regulated by fibroblasts and endothelial cells that produce collagen, and collagenase that act to degrade collagen. Numerous other enzymes exist that either promote or inhibit the activity of collagenase. In keloids, the balance of these molecules is in the favor of unchecked collagen production and tardy removal [14, 15].

Another factor of keloid development is the mechanical tension exerted on wounds. Mechanical tension encourages the proliferation of fibroblasts and synthesis of collagen [16] [17]. In addition, the vector of tension dictates the alignment of collagen fibers [18]. In general, the orientation of newly produced collagen fibers tends to be perpendicular to the direction of muscle contraction. This is why surgeons are taught to place incisions along the Langer skin lines of tension, which are perpendicular to the muscle fibers and often parallel to natural skin creases. Thus, wounds that do not conform to the Langer lines are expected to undergo unsightly scarring and higher occurrence of keloids.

Multiple treatment strategies have been published in the literature, none of which guarantees complete treatment on their own. Surgical excision is an option, although without adjunct therapy, it almost invariably results in recurrence. Either total or subtotal excision can be performed, and intradermal or subcuticular wound closure with monofilamentous rather than braided suture is preferred to minimize local inflammation [19]. Radiation therapy immediately after surgical excision is known to effectively reduce recurrence rates by causing apoptosis of keloid fibroblasts [20]. Radiation therapy, however, is contraindicated in children, pregnant women, and visceral areas, and the theoretical risk of carcinogenesis limits its use in certain patients. Silicone gel or sheets, when applied for over 12 hours each day, can also be effective in the treatment or prevention of keloids. Silicone is relatively inert, and the impermeable membrane enhances keratinocyte hydration, which in turn elicits growth factors that regulate fibroblast function [21]. Silicone gel and sheets rarely accompany side effects, but their effectiveness is critically dependent on patient compliance. Other therapies include cryosurgery, 5-FU, interferon, bleomycin, retinoids, imiquimod, verapamil, and combinations of these drugs with TAC [22].

Intralesional corticosteroid injection is perhaps the most widely used treatment for keloids. Corticosteroids are effective vasoconstrictors, depriving keloid tissue of oxygen and nutrients. In addition, they have antimitotic effects, suppressing the proliferation of keratinocytes and fibroblasts. They also enhance the production of collagenase, lower levels of collagenase inhibitors, promote collagen degeneration, and inhibit leukocyte migration and phagocytosis [23, 24]. The most common type of corticosteroid used for scar treatment is TAC. The response rate to TAC injection is variable, however, ranging from 50 to100%. According to the literature, the recurrence rate after TAC injection was 33% and 50% after 1 and 5 years, respectively [25]. 5-year recurrence rates for keloid excision followed by TAC ranged from 8% to 50% [6]. In a 10-year study of keloids and hypertrophic scars conducted by Darzi et al., TAC injection alone led to symptomatic improvement in 72% of patients and complete flattening in 64% of lesions [26]. Acosta et al. reported in their prospective clinical trial with pediatric patients an 82.7% reduction in size of keloid lesions between first and last TAC injections (*p *< 0.001) [27]. They also concluded that the clinical response to treatment was not associated with lesion site, age, or etiology. There is much debate with regard to the optimal dose and interval of TCA injection in the treatment of keloids. One study compared 7.5 mg/cm^2^ of TCA injection to 15 mg/cm^2^ at an interval of 4 weeks and concluded the former produced more favorable results with fewer complications [28]. Coppola and colleagues summarized in their review paper the various TCA injection doses and intervals that were adopted by different authors [29]. The dose ranged from 1 to as large as 20 mg/cm^2^, and the concentration from 4 to 40 mg/mL. The majority of authors selected interval of 4 weeks. The subjects of this study were treated with a rather lower dose of 4 mg/cm^2^, considering that they all had their keloid lesions surgically removed beforehand. In other words, the objective of TCA and laser therapy was rather focused on preventing recurrence and thus a low dose of TCA was deemed sufficient. The side effects of intralesional corticosteroid injection include telangiectasia, skin and fat atrophy, hypopigmentation, and in rare cases, Cushing’s syndrome. The risk of local complication increases as injections are inadvertently extended to nearby healthy tissues [24]. Cushing’s syndrome has been reported in adults [30] as well as in children [31], and close monitoring for its signs and symptoms is recommended.

There are various ablative and non-ablative laser devices that can be utilized to treat keloids [32]. Ablative lasers are absorbed by water content in the skin and remove the epidermis. Examples include erbium-doped yttrium aluminum garnet (Er:YAG) and carbon dioxide (CO2) lasers. These lasers can produce remarkable therapeutic effects with regard to keloids and hypertrophic scars, but complications such as pain, exacerbation of erythema, and hyper- and hypopigmentation limit their use [33]. Non-ablative laser has the advantage of preserving the epidermis, resulting in shorter downtime [25]. These include potassium titanyl phosphate (KTP), pulsed dye laser (PDL), and Nd:YAG lasers. The 1064 nm Nd:YAG laser reaches the lower dermis and is absorbed by melanin, hemoglobin, and to a lesser extent, water. This laser has been used in the treatment of keloids since first reported by Abegel in 1984 [10, 34]. The heat energy produced by the laser is thought to promote collagen denaturation and realignment of collagen fibers [35, 36]. The Nd:YAG laser penetrates the dermis more deeply than the pulsed dye laser and thus could be a more preferable option in the treatment of thick keloids and hypertrophic scars. In a systematic review of the use of Nd:YAG laser in keloid treatment, the Nd:YAG laser effectively reduced the VSS of keloids, though the effect was generally smaller than in hypertrophic scars [37]. Akaishi and colleagues reported the efficacy of long pulsed 1064 nm Nd:YAG laser with low fluence (14J/cm^2^) in the treatment of keloids and hypertrophic scars [38]. Such energy density was not only sufficient to reduce erythema, hypertrophy, hardness, itching, and pain, but also changed the tissue collagen structure on histologic evaluation and electron microscopy. Likewise, a low fluence was selected for the patients of this study, for whom the laser was an adjunct therapy to surgery and TCA injection. Each lesion was treated with 6 passes based on previous literature [39].

Combination therapy may be more effective than a single mode of treatment in reducing keloid recurrence. Kumar and colleagues demonstrated that keloid patients who were resistant to treatment with Nd:YAG laser eventually underwent complete resolution with the addition of intralesional TAC [40]. In a retrospective study performed by Rossi and colleagues, the combination of 1064 nm Nd:YAG laser and intralesional TAC resulted in reduced thickness and erythema of the keloid scars compared to intralesional TAC alone [41]. The synergistic effect of laser and TAC was also explored by Kraeva and colleagues, who conceptualized the laser-assisted delivery of topical TAC. According to the authors, the CO2 laser enhanced the uptake of TAC by the dermis and reduced the injection pain. Conversely, the steroid alleviated the post-inflammatory hyperpigmentation due to laser procedure [42]. In addition, Stucker et al. reported that intralesional TAC injection hampers the recurrence of keloids treated with the CO2 laser [43]. Garg et al. concluded that patients who did not comply to regular checkups for TAC injection after laser therapy exhibited higher recurrence rates compared to those patients who were treated regularly with TAC [44].

The results of our study demonstrate that patients who were treated with dual therapy combining TAC injection with Nd:YAG laser experienced a greater improvement in the mean POSAS score at 12 months after operation compared to those who received TAC injections alone. While there was no significant difference in the mean mVSS score between the two groups at both 6 and 12 month follow-ups, statistically significant disparities in recurrence rates were observed. Despite the potential subjectivity in POSAS scores, the combination therapy group showed superior outcomes in managing keloid scars compared to monotherapy. Additionally, the absence of complications supports the safety of these treatments.

Our study was limited by the relatively short follow-up period that did not exceed 2 years. Unlike previous studies that directly compared the combination of TAC and laser to TAC-only treatment, all the patients who enrolled in our study underwent initial surgical excision of their keloid lesions. Since surgical treatment is usually reserved for patients with more severe keloids, the conclusions of our research may not be accurately applied to patients with milder lesions. In the same vein, keloids that were thick enough as to necessitate surgical excision would have benefitted more from the add-on therapy with Nd:YAG laser, which has good penetration into the lower dermis. This study did not incorporate a group that only received Nd:YAG laser therapy, as did the study by Rossi et al [41]. Our study design was based on the presumption that TAC injection be the mainstay treatment for keloids and thus every subject was injected with TAC. In other words, the main objective of our study was to explore the possibility of synergy between these two treatments, rather than to assess the effect of Nd:YAG laser on keloids. Although we did not include a ‘laser only’ group in the study design, we believe this research made progress in the field of keloid management. First, we had a fairly large sample size of 111 patients, while previous studies either involved small samples [41, 43, 44] or were a case study [42]. Moreover, we adopted extensive evaluation criteria that included not only objective factors such as erythema and scar thickness, but also subjective factors such as patient satisfaction. Nevertheless, the precise mechanism of the potential synergy of TAC and laser therapy remains ambiguous and calls for future in vitro and in vivo studies.

## Conclusion

Dual therapy involving 5 monthly sessions of TAC injection combined with *Q*-switched 1064 nm Nd:YAG laser treatment was superior to TAC injection alone in reducing keloid recurrence at 6 and 12 months and improving keloid scar grade at 12 months following surgical excision. The combination therapy also demonstrated improvement in patient-reported outcomes, confirming its effectiveness and safety for keloid treatment.

## Abbreviations

Nd:YAG: Neodymium-doped yttrium aluminum garnet
TAC: Triamcinolone acetonide injection
mVSS: Modified Vancouver Scar Scale
POSAS: Patient and Observer Scar Assessment Scale
TGF-β: Transforming growth factor-β
PDGF: Platelet-derived growth factor
ECM: Extracellular matrix
5-FU: 5-Fluorouracil
